# Human Germline Antibody Gene Segments Encode Polyspecific Antibodies

**DOI:** 10.1371/journal.pcbi.1003045

**Published:** 2013-04-25

**Authors:** Jordan R. Willis, Bryan S. Briney, Samuel L. DeLuca, James E. Crowe, Jens Meiler

**Affiliations:** 1 Center for Structural Biology, Vanderbilt University, Nashville, Tennessee, United States of America; 2 Institute for Chemical Biology, Vanderbilt University Medical Center, Nashville, Tennessee, United States of America; 3 Department of Chemistry, Vanderbilt University, Nashville, Tennessee, United States of America; 4 Department of Pathology, Microbiology and Immunology, Vanderbilt University Medical Center, Nashville, Tennessee, United States of America; 5 Department of Pediatrics, Vanderbilt University Medical Center, Nashville, Tennessee, United States of America; 6 Department of Pharmacology, Vanderbilt University Medical Center, Nashville, Tennessee, United States of America; La Jolla Institute for Allergy and Immunology, United States of America

## Abstract

Structural flexibility in germline gene-encoded antibodies allows promiscuous binding to diverse antigens. The binding affinity and specificity for a particular epitope typically increase as antibody genes acquire somatic mutations in antigen-stimulated B cells. In this work, we investigated whether germline gene-encoded antibodies are optimal for polyspecificity by determining the basis for recognition of diverse antigens by antibodies encoded by three V_H_ gene segments. Panels of somatically mutated antibodies encoded by a common V_H_ gene, but each binding to a different antigen, were computationally redesigned to predict antibodies that could engage multiple antigens at once. The Rosetta multi-state design process predicted antibody sequences for the entire heavy chain variable region, including framework, CDR1, and CDR2 mutations. The predicted sequences matched the germline gene sequences to a remarkable degree, revealing by computational design the residues that are predicted to enable polyspecificity, *i.e.*, binding of many unrelated antigens with a common sequence. The process thereby reverses antibody maturation *in silico*. In contrast, when designing antibodies to bind a single antigen, a sequence similar to that of the mature antibody sequence was returned, mimicking natural antibody maturation *in silico*. We demonstrated that the Rosetta computational design algorithm captures important aspects of antibody/antigen recognition. While the hypervariable region CDR3 often mediates much of the specificity of mature antibodies, we identified key positions in the V_H_ gene encoding CDR1, CDR2, and the immunoglobulin framework that are critical contributors for polyspecificity in germline antibodies. Computational design of antibodies capable of binding multiple antigens may allow the rational design of antibodies that retain polyspecificity for diverse epitope binding.

## Author Summary

Human antibodies are critical for eradication of viral and bacterial infections, while providing the basis for immunological memory. Antibody protein molecules are encoded by several recombined germline gene segments prior to antigen exposure. The initial set of antibodies that are generated by recombination in the bone marrow is the antigen-naïve antibody repertoire. It is of great interest to know how a finite set of such germline gene-encoded antibodies can recognize the large number of possible foreign antigens. A current hypothesis in the field suggests that antibodies encoded by germline gene segments are structurally flexible and able to accommodate binding to many antigens, much like one glove fitting the shape of many hands. The phenomenon of one structure binding to many targets is known as polyspecificity. Here we further support this hypothesis by showing that entire antibody protein variable regions encoded by germline gene segments are close to ideal for polyspecificity. We used computational design algorithms to explore antibody sequence space rapidly and predict optimal sequences to achieve polyspecificity. The resulting designed sequences recapitulated the germline gene segment sequences and highlighted residues critical for achieving polyspecificity. These results suggest how a finite set of antibody germline gene segments can encode antibodies that can engage a large number of antigens.

## Introduction

Antibodies are the primary effector molecules in the humoral immune system, inhibiting pathogenicity of microbes by binding to surface-exposed elements on foreign particles [1]. Antibodies are encoded by the rearrangement of variable (V), diversity (D), and joining (J) gene segments into recombined genes that encode a large but ultimately finite number of unmutated antibody structures, known as the germline repertoire [2]. There are approximately 10^4^ combinations of the V, D, and J heavy chain gene segments and an estimated 10^11^ possible combinations when junctional diversity is considered [3]. This number of potential antibodies is far less than the number of epitopes on foreign antigens to which one could be exposed. The germline gene repertoire therefore encodes a finite number of starting structures in the germline repertoire that must be capable of recognizing a large and diverse array of antigens [3]–[5]. The immense breadth of binding of the antibody repertoire is achieved by structural diversity in the antigen-binding site.

The lock-and-key model of binding between two rigid proteins, dominated the antibody field for many years [6], [7]. Somatic mutations acquired during affinity maturation refine the pre-bound structure of a specific antibody to bind optimally to a single particular antigen. In contrast, the conformational flexibility hypothesis suggests that germline gene-coded antibodies retain a degree of structural plasticity in their backbone in order to bind a number of different unrelated antigens, a capacity referred to here as polyspecificity. Although polyspecificity has been demonstrated in a variety of biochemical and structural studies, the molecular mechanism that antibodies use to achieve polyspecificity remains poorly understood [4], [8]–[10]. For antibodies, a large body of work has attributed polyspecificity to the nature of their germline gene sequences. It has been reported that polyspecific antibodies often retain a larger proportion of germline gene sequences than more specific antibodies [6], [11]–[13].

The conformational flexibility concept in protein binding suggests that an unbound protein assumes a variety of conformations, a subset of which is recognized by the interacting partner. The induced-fit model hypothesizes that upon binding conformational changes are induced to accommodate the interacting structure (reviewed in [6], [9]). Originally introduced as an extension to the lock-and-key model to explain conformational changes in presumably rigid structures upon interaction, the induced fit model can also be combined with the conformational flexibility concept to allow for additional small conformational changes in the interacting subset of structures [14], [15]. Conformational flexibility is emerging as an important hypothesis to explain polyspecificity and changes in affinity between germline and mature antibody sequences [3], [6]–[10], [16]–[28]. Kinetic experiments with antibodies show a triphasic distribution that, in some cases, appears to reflect the existence of multiple conformations of the unbound antibody in solution [9]. More recently, structural studies along with computational tools have corroborated these findings by showing that antibodies encoded by germline gene sequences retain flexibility in their CDR3 loops [24], [29]. For example, Babor *et al.* redesigned germline or mature CDR3 loops in antibodies that had been crystallized in free or antigen-bound states [29]. These investigators found that germline gene-encoded CDR3 sequences are nearly optimal for conformational flexibility. The study, while exceptional in its concept, was limited as the dataset contained many antibody/hapten complexes, which may not reflect the biology of interactions with larger protein targets that are more typical in foreign pathogens. Some antibodies classified as “germline” in the study were not from antigen-naïve cells. Further, that study exclusively analyzed the CDR3 loop, not the entire variable region.

Schmidt *et al.* have recently used molecular dynamics simulations and structural analysis to determine how mutations in the antibody variable domain enhance antigen binding to influenza HA [30]. In the study, they found two broadly neutralizing antibodies that have branched in lineage from a common intermediate, and an unmutated common ancestor (UCA) in which they obtained high-resolution crystal structures. They found that even though the UCA and mature antibodies have nearly identical binding configurations, the affinity for influenza for the mature antibodies was 40-fold greater than the UCA. Molecular dynamics simulations predicted that the paratope in unbound UCA was not in an optimal conformation for binding, while the mature antibodies had a higher probability of being pre-configured for the influenza HA epitope.

The present study complements the work of Babor *et al.* and addresses some of the limitations by focusing on a limited set of germline V_H_-genes that are commonly used in antibodies that bind diverse antigens. The V_H_-gene encodes the CDR1 and CDR2 loops and much of the immunoglobulin framework regions but not the CDR3 loop. We hypothesized that the conformational flexibility mediating the polyspecificity of germline gene-encoded antibodies is determined at least in part by the heavy chain variable region encoded by the V_H_ gene. The focus of the current study was to test this hypothesis using computational design. Specifically, we analyzed the somatic mutations in sets of mature antibodies that derived from the same V_H_ gene and for which co-crystal structures with biologically relevant target proteins were available.

Sets of mature antibody-antigen complexes incorporating antibodies that derived from a common germline V_H_ gene were input into the Rosetta ‘multi-state’ design algorithm that recovers the optimal single sequence for an antibody to bind all antigens simultaneously [29], [31], [32]. The sequences recovered were considered inherently flexible, since they were predicted to accommodate binding to diverse antigens using a structurally diverse set of antibody conformational states. Remarkably, the derived sequences matched the germline V_H_ gene sequence to an unexpectedly high degree. Further, we found that even residues within the antibody framework distal from the antigen-antibody interface were critical for polyspecificity.

In contrast, when each antibody was designed for binding to a single antigen, using a ‘single-state’ design approach, the mature sequences of antibodies were recovered, suggesting that mature antibody sequences are optimal for binding to a selected antigen and preferred over the germline gene-encoded sequence. The changes in recovered sequences between the multi-state design protocol that considered polyspecificty and the single-state design that considered monospecificity appeared to recapitulate *in silico* the *in vivo* process of somatic hypermutation and selection.

Fundamentally, our approach compares germline and mature antibody sequences optimized in nature through evolution and maturation with sequences predicted to be optimal based on Rosetta's energy function applied to a set of co-crystallized antibody/antigen complexes. The power of the present approach is that we predicted germline and mature sequences *in silico* without any prior knowledge of either, which is an important step towards rational antibody design. We discuss several important assumptions and limitations, including the assumption that the Rosetta design protocol determines the optimal sequence for any given design challenge, that the conformational space of the germline gene-encoded antibody is described by a finite set of co-crystal structures (finite ensemble bias), that the germline antibodies were able to adopt the conformations of each of the mature antibodies derived from it, and that the germline and mature sequences observed in nature are optimal for polyspecificity and high affinity (evolutionary sequence bias), respectively. Summarizing these considerations we expect imperfect agreement of *in silico* predicted and natively observed mature and germline antibody sequences. Nevertheless, we found statistically significant trends that were derived from a large number of example cases that supported the ‘conformational selection’ paradigm inherent to the conformational flexibility hypothesis for germline gene-encoded antibody interactions with target antigens, *i.e.*, these antibodies exist in a large number of conformations in the antigen-unbound state. We expect that results of this type of analysis will continue to improve as the size of the collection of conformational ensembles available in the Protein Data Bank (PDB) increases and as the accuracy of the Rosetta energy function continues to improve.

## Results

### Multi- and Single-State Design of Antigen-Antibody Complexes

**Figure 1:**
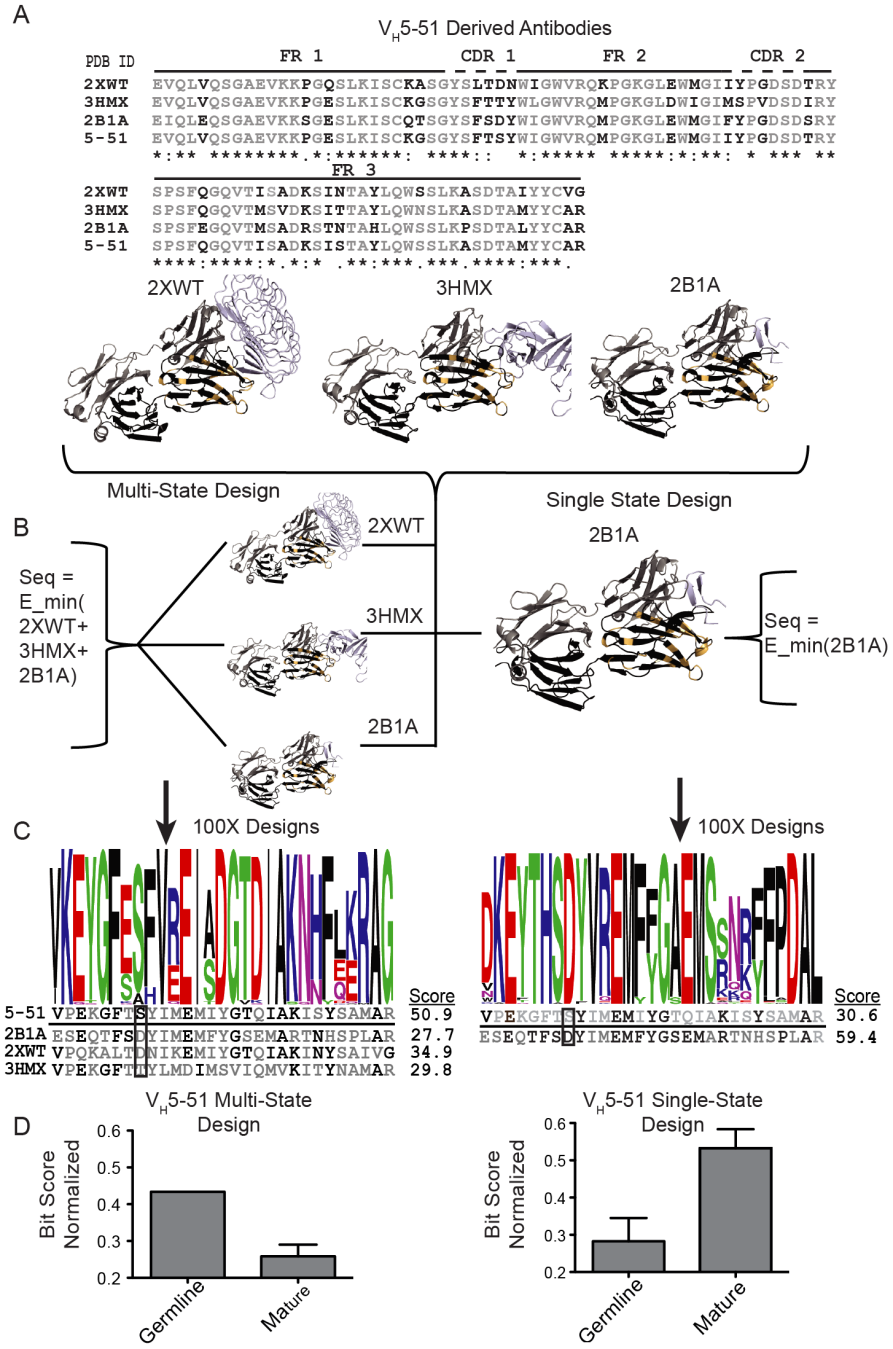
Multi-state and single-state design methodology. For a simplified view of the methodology, only the complexes from inferred germline V_H_5-51 are presented. (A) The heavy chain variable segment amino acid sequences taken from the PDB were aligned. Position candidates were chosen for design if the position differed from the germline sequence in at least one mature complex. (B) Co-crystal structures for each V_H_5-51 derived complex are shown with heavy chain in black, light chain in grey, antigen in magenta, and designed positions highlighted in gold. Single- and multi-state design schemes are shown where each complex was designed alone (single-state) where the designed positions were optimized to minimize the energy for a single antigen target, or a minimized energy for all complexes considered together (multi-state) where the sequence returned was an energetic consensus among each complex considered. (C) Sequence logos were generated to show 100 design models. Each position in the sequence logo corresponds to a position conserved for design. The sequence logos then were compared to the mature or germline sequence for each antibody. (D) Bit-scores were determined quantitatively by measuring the frequency of a letter at each position. The bit-score measures the designed residues compared to either the germline sequence or the mature sequence. The normalization factor was the [total bit-score]/[perfect design score](see Methods).

We compiled panels of antigen-antibody complexes from the Protein Data Bank (PDB) in which the antibody heavy chain variable region was encoded by germline V_H_ genes, designated V_H_3-23, V_H_1-69, or V_H_5-51 [33], [34]. Antigen-antibody complexes were selected if and only if they contained *Homo sapiens* or humanized antibodies and the binding partner was a protein antigen. No further selection criteria were introduced as the exact nature of the antigen and antibody isotype where not considered. The exhaustive search of the PDB returns 10, 8 or 3 candidate complexes for V_H_1-69, V_H_3-23, or V_H_5-51 respectively (Table 1). For each panel we compared the mature (somatically mutated) sequence to the inferred germline gene sequence via a multiple sequence alignment (Figure 1A, S1). The number of mutations with respect to the germline sequence range from 4 to 23 mutations with an average of 12.2. All CDR1, CDR2, and framework positions that differed from the germline sequence of the common V_H_ gene sequence in at least one position in the multiple sequence alignment were included in the computational design simulations as “variable positions”. Note that the present study explicitly excluded positions that remained unchanged as no claims can be made with respect to the relevance of these positions for conformational flexibility or polyspecificity. Our analysis is limited to antibody regions encoded by the V-gene as only this region can be unambiguously aligned within each set of antibodies. Therefore, we excluded D-gene and J-gene that encode CDRH3, and antibody light chain from the present analysis.

**Table 1:** Antibody-antigen test set complexes.

PDB ID	V_H_ * Germline	Antibody	Ligand	V_H_ * Mutations
2CMR	1-69*01	D5	gp41	6
3FKU	1-69*01	F10	HA	13
3GBM	1-69*01	CR6261	HA	15
3MA9	1-69*01	8066	gp41	4
3MAC	1-69*01	8062	gp120	7
3P30	1-69*01	1281	gp41	20
1G9M	1-69*02	17b	gp120	21
2DD8	1-69*05	M396	SARS-RBD	5
2XRA	1-69*05	HK20	gp41	14
2XTJ	1-69*10	1D05	PCSK9	4
2QQN	3-23*01	anti-Nrps-1	Nrps-1	10
2R56	3-23*01	IgE	BLG	23
2VYR	3-23*01	VH9	MDM4	10
3KR3	3-23*01	DX-2647	IGF-II	8
1S78	3-23*04	Pertiuzimab	ErbB2	22
2FJG	3-23*04	G6	VEGF	15
3DVN	3-23*04	Apu2.16	Ubiquitin	18
3BN9	3-23*04	E2	MT-SP1	5
2B1A	5-51*01	2219	UG1033	17
2XWT	5-51*01	K1-70	TSHR	8
3HMX	5-51*01	Ustekinumab	IL-12	12

Details of the 10, 8, and 3 complexes for V_H_1-69, V_H_3-23, and V_H_ 5-51 respectively. The antibodies bind a diverse set of antigens but each share a common germline across a test set. The V_H_ mutation count of amino acid mutations away from their inferred germline gene.

* Inferred germline sequence and mutations predicted from IMGT/3Dstructure-DB [45].

**Figure 2:**
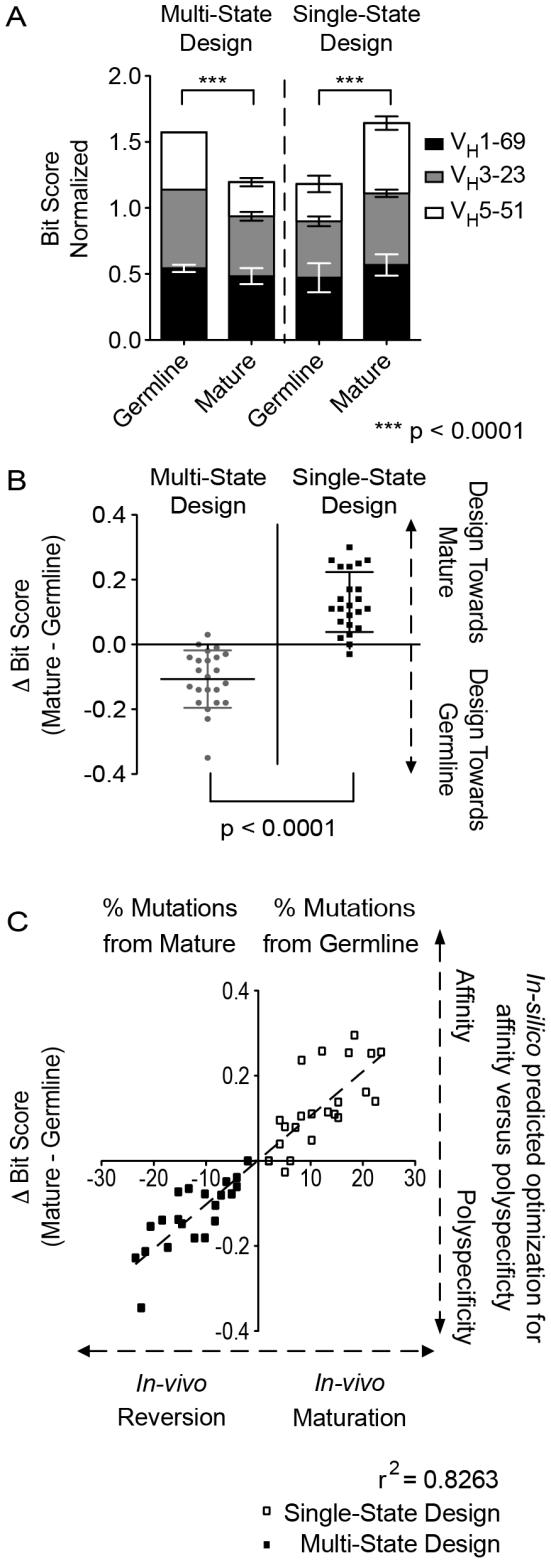
Multi-state designs toward the germline sequence, single-state to mature sequences. Antibodies encoded by the same inferred germline V_H_ gene preferred germline sequences when considered in the multi-state design, inferring a more flexible combining site. (A) The bar graph shows the bit-score for each of the three different inferred germline groups and then the sum of the scores in a grouped bar. A perfect design would have a normalized bit-score of 1.0, and summated score of 3.0 for three germline groups. Multi-state design preferred germline sequences for all complexes, while in contrast single-state design preferred mature sequences (p<0.0001). (B) The change in bit-score is determined to be the proclivity to either the mature (positive score) or the germline (negative score) sequence. Each complex was assigned a change in bit-score. The change in proclivity between design protocols was significant (p<0.0001). (C) Each complex was scored against mature and germline sequences and a difference was calculated (Δbit-score). Positive numbers returned showed a proclivity towards mature sequences, while a negative score suggested a design toward germline. A tight correlation was observed (r^2^ = 0.8263) for the *in silico* predicted optimization for specificity versus polyspecificity *(*Δbit-score) and the *in vivo* maturation process (plotted as the mutation percentage away from V_H_ gene sequence).

The fixed-backbone design algorithm of Rosetta [35] simultaneously samples amino acid identity and conformation in all variable positions to identify the sequence and conformation that return minimal energy for the given protein backbone of the antibody/antigen complex. In the present experiment, we used multi-state design [32] to find a single sequence that minimized energy with all antigens within each V_H_ gene-encoded group (Figure 1B). To reduce noise in the outcome of the computations, 100 simulations were executed, and results are displayed using WebLogo representation [36] (Figure 1C). For instance, position 31 (PDB numbering, boxed in Figure 1C) in the sequence alignment of antibodies encoded by V_H_5-51 diverged from a germline serine residue in the sequence for all three complexes. Complexes 2B1A and 2XWT (PDB code) possess an aspartate residue in this position acquired by somatic mutation, while 3HMX has a threonine in the same position. The multi-state design protocol selected the germline residue serine as the energetically most favorable residue out of all 20 possible genetically encoded amino acids when interaction with all three structurally diverse antigens is required (Figures 1C and S2-C). The experiment was repeated as three separate ‘single-state design’ experiments (Figure 1B) to predict the sequences and conformations that minimized interaction energy for each antigen individually. The resulting sequences were compared to both the inferred germline and the mature sequence (Figure 1C). In this experiment position 31 is predicted as an aspartate for complexes 2B1A (Figure 1C) and 2XWT, and as a threonine for 3HMX, the mature amino acid sequence (data not shown).

For quantitative analysis, the bit-score was used to designate each position as either being reverted towards germline sequence, recovered as the mature sequence, or neither. Each design outcome is compared to the mature or germline sequence, respectively, by computing a bit-score ‘recovery’ measure. Schneider and colleagues, who extended the work of Claude Shannon, showed that bit-scores contain information about the sequence position if each amino acid at that position is not equi-probable [37], [38]. The advantage of the bit-score measure in comparison to a more simplistic percentage-recovery is that it analyzes the relative probabilities of all twenty amino acids in a particular sequence position, not just the probability of the correct one. It thereby arrives at an accurate measure of ‘surprise’ of seeing a certain outcome, a normalized measure in information theory that can be readily compared between different experiments. In our experiment high bit-score for the germline sequence indicated that among the 100 designed sequences, germline gene-encoded residues were chosen in a large number of instances (Figure 1D). To facilitate comparison across complexes, we determined the sum bit-scores over all designed positions and normalized the score to fall between 0 and 1 by division with the maximum bit-score that could be achieved, *i.e.*, every amino acid position designed towards a germline or mature sequence. For a more detailed explanation of the bit-score metric and protocol, see Methods.

### Specificity Inferred by Sequence Design

The results of the multi-state design simulations returned sequences that resembled germline gene-encoded sequences more often than mature sequences. This finding was remarkable as no information about germline sequences was input into the simulation. We found that the designed sequences gave normalized bit-scores of 0.54, 0.60, and 0.43 for germline genes V_H_1-69, V_H_3-23, or V_H_5-51 respectively. In contrast, statistically significant reduced bit-scores of 0.48, 0.45, or 0.26 (p<0.0001) were observed when comparing the designed sequences with the mature genes (Figures 2A, S2 and S3).

The single-state redesign of mature antibodies for binding to their associated antigen gave normalized bit-scores of 0.47, 0.43, or 0.28 for comparison with germline gene-encoded sequences and 0.57, 0.54, or 0.53 for comparison with mature sequences of V_H_1-69, V_H_3-23 or V_H_5-51, respectively. In this design experiment a proclivity to recover the somatically mutated mature sequences was observed (Figures 2A and S3). Given that a normalized bit-score is the preference for each design experiment to match a certain sequence profile, a high bit-score to germline sequence indicates the output matching the germline profile, while a high bit-score to the mature sequence indicates a preference for the mature profile, each design experiment outcome can be measured as a difference in bit-scores (mature - germline). With this definition, a preference for mature sequence gave a positive Δbit-score, while a preference for germline residues gave a negative Δbit-score for a given complex – *i.e.*, the Δbit-score provided an *in silico* predicted metric for antibody optimization for affinity to a specific antigen versus polyspecificty. We observed positive values for single-state design and negative values for multi-state design, indicating a preference for the mature or germline sequences, respectively (Figure 2B, p<0.0001).

### *In Vivo* Affinity Maturation correlates with *In Silico* Predicted Optimization for Affinity versus Polyspecificity

The number of somatic mutations can be used as a measure of the maturity of an antibody [39]. Hence, we asked the question if the Δbit-score, the change in proclivity for a germline or mature sequence, correlated with affinity, *i.e.*, if tendency to recover mature versus germline sequences increased as antibody maturation progressed. Such a correlation would indicate that as antibodies mature, features of the germline sequence critical for polyspecificity are replaced with features critical to recognize one target antigen. Figure 2C shows the somatic mutation percentage of antibodies in each complex as a metric for *“in vivo* maturation” correlated with the Δbit-score as a metric for *“in silico* predicted optimization for affinity versus polyspecificty”. For positive Δbit-scores, the mature sequence was preferred, indicating a preference for specificity. For negative values, the germline sequence, and hence polyspecificity was preferred. The correlation coefficient for the “*in vivo* affinity maturation” and “*in silico predicted optimization for affinity vs. polyspecificity”* was 0.83.

The correlation coefficient increased to 0.89 when considering only antibodies with more than 12 somatic mutations from their inferred germline gene sequence. The change in proclivity for germline sequence in multi-state design and mature sequence in single-state design became more pronounced (Figure S4).

### Backbone Conformational Space was Increased at Positions that Reverted to the Germline Sequence in Multi-state Design

**Figure 3:**
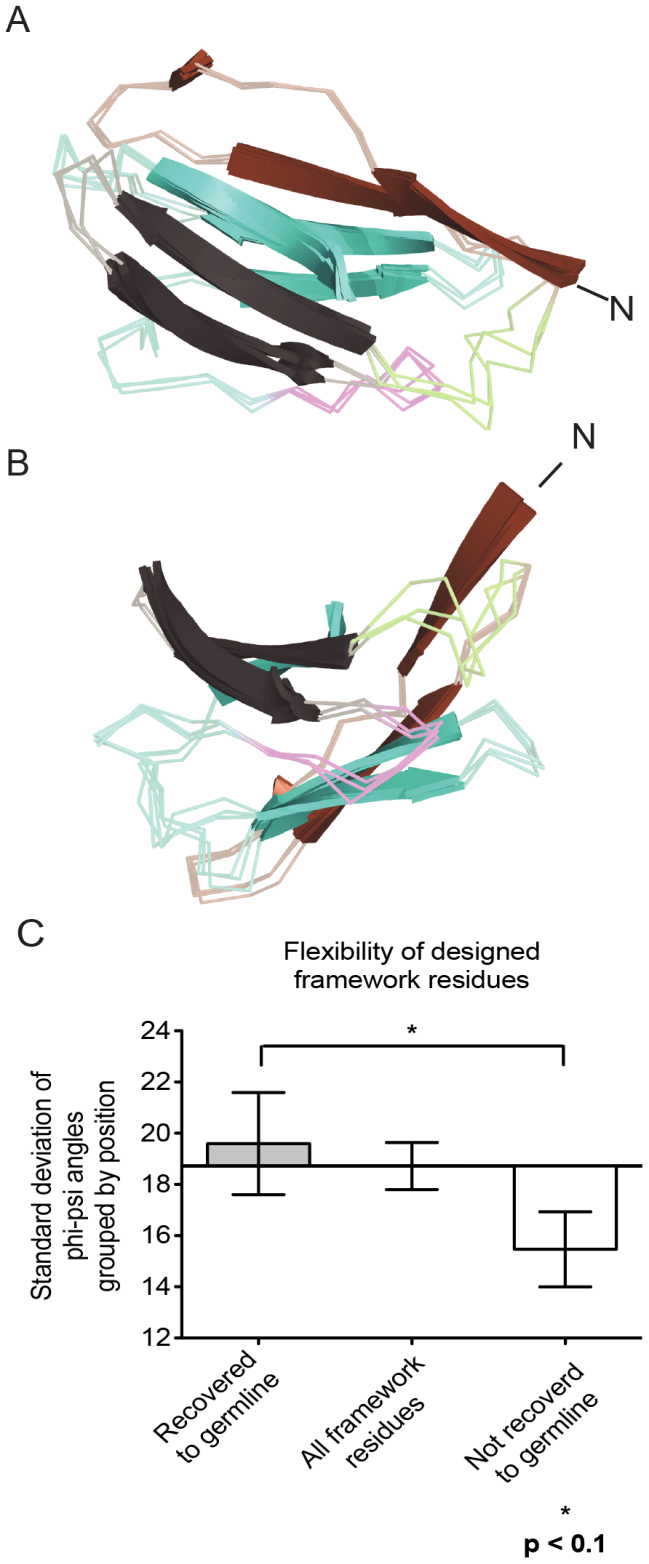
Phi-psi variances for framework residues. The degree of structural variation of the framework residues were measured as the standard deviation of the phi and psi angles over each residue position. (A) Side view of immunoglobulin fold for V_H_5-51 complexes aligned by framework residues. Beta-sheets included in the analysis are shown as a cartoon representation, while loop regions are in a transparent ribbon representation. Framework 1 is shown in brown, CDR 1 in green, framework 2 in black, CDR 2 in magenta, and framework 3 in cyan. (B) Top down view. (C) The standard deviations of the phi-psi angles of each framework position were binned into either a residue that was found to be critical for polyspecificity (recovered to germline) or a residue that was not recovered to germline in multi-state design. For each position, the phi-psi angles were averaged, and the standard error of the mean was calculated. An average of 19.6°±2.0° for germline recovered residues and 15.47°±1.5° for non-germline recovered residues supporting our hypothesis that residues which enable polyspecificity alter beta-sheet packing to a greater degree than residues that do not. The axis is normalized to 18.7°±0.9°, the average deviation for all beta-sheet framework positions.

Torsional phi-psi angles in the protein backbone were compared across the sets of experimental structures for positions that recovered to germline sequence for multi-state design and those positions that recovered to a non-germline sequence. We found that positions that converted back to germline in multi-state design, *i.e.*, positions critical for conformational flexibility according to the simulation, had a deviation of 19.6°±2.0° across beta-sheet phi-psi torsion angles. Sequence positions that did not recover to a germline gene-encoded amino acid had a statistically significant (p = 0.099) reduced deviation of 15.5°±1.5° for beta-sheet backbone torsion angles (Figure 3). Considering the limited range for beta-sheet backbone torsion angles, we don't expect large deviations. For reference, all framework residue beta-sheets in antibody-antigen complexes across our dataset have an average phi-psi deviation of 18.7°±0.9° (see methods).

### Impact of Residue Interface Location and Burial on Optimization for Affinity Versus Polyspecificity

**Figure 4:**
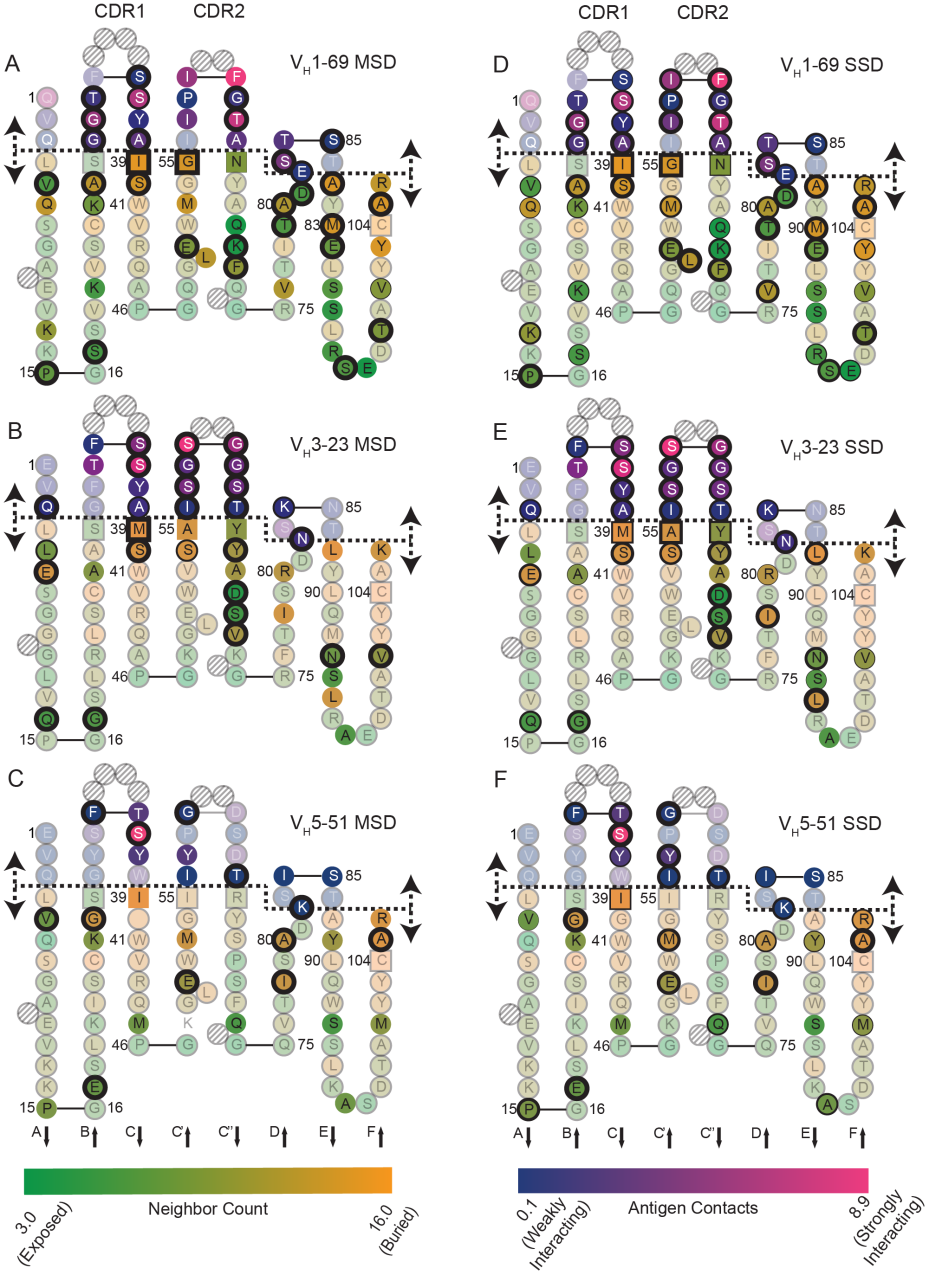
Modified Colliers de Perles representation of V_H_ gene segments. The 98 amino acids present in V_H_ 1-69, V_H_3-23, or V_H_5-51 are shown in a Collier de Perles two-dimensional representation and numbered according to the IMGT numbering scheme [40]. Hatched circles are missing residues according to the IMGT numbering scheme and are shown to make graphs consistent. Square boxes represent the boundary between framework and CDR loops. The anti-parallel beta-sheets are represented A–F. A dashed line is shown that divides interface residues with residues that were found to be outside of the interface in a majority of the cases. Interface residues are colored with a blue-pink gradient indicating a numerical antigen contact score defined by a change in neighbors between the free and bound complex (see Methods). Non-interface residues are colored with a green-orange gradient according to their degree of burial defined through a neighbor count. Residues that are transparent were not considered for redesign as they were conserved across all complexes considered. (A, B, C) show the germline sequence represented in the immunoglobulin fold with the thickness of each line representing the design bit-score for that position relative to the germline sequence for multi-state design protocols for V_H_1-69, V_H_3-23, or V_H_5-51, respectively. (D, E, F) show the germline sequence represented, but the thickness of the line corresponds to the mature sequence bit-score averaged over each complex for the single-state design protocol for V_H_1-69, V_H_3-23, or V_H_5-51, respectively.

Figure 4 maps each amino acid position encoded by the V_H_ gene segment onto the immunoglobulin fold using a custom Collier de Perles representation, as described by Ruiz and Lefranc [40]. We modified the output to distinguish positions by location in the interface with the antigen and the degree of burial. We correlated these metrics to the bit-score at a per-residue level. Each residue given is in IMGT numbering.

For multi-state design (Figure 4A–C), 33 out of a possible 46 of the designed interface residues (72%) contributed to polyspecificity, *i.e.*, recovered to germline sequence with a normalized bit-score>0. Remarkably, also 41 out of 77 residues outside the interface (53%) recovered to germline. Residues 25, 40 and 105, far removed from the interface, recovered perfectly (normalized bit-score = 1) in at least two of the three germline gene test sets. These residues are highly buried, with a neighbor count score of 13.3±0.5. The intermediately packed residues 17, 51, 70, and 71, with an average neighbor score of 8.6±2.2 neighbors, were predicted to contribute to polyspecificity, even though they lie in distal positions from the antigen-binding site. The interface residues 35, 63, 64, and 82 were found to contribute to polyspecificity in two out of the three germline gene test sets. A conserved serine, which was found in all three germline sequences at position 36 in the CDR1, was the only residue identified as critical for polyspecificity in all three germline genes.

In contrast, for single-state design, it is more difficult to deduce overall trends for any specific position as the paratope is altered in each antibody and the recognized epitopes cover diverse structural space. Generally, when each complex was considered individually, 214 designed interface residues recovered to their mature sequence out of a possible 340 designed amino acids, indicating their importance for recognition of, and affinity for binding to, the specific antigen (63%, Figure 4D–F). When non-interface residues were considered, 411 out of a possible 699 designed residues recovered to their mature sequence (59%).

We also examined 255 designed residues that were found to be somatically mutated away from residues encoded by their inferred germline gene. Of these mutations, 68 out of 110 interface residues recovered to the mature sequence (62%) in single-state design, while 72 out of 145 non-interface residues recovered to the mature sequence (49%).

Residues that were found to be critical for polyspecificity, *i.e.*, reverted to germline in multi-state design, differed substantially for each germline gene test set considered. For the V_H_1-69 gene derived antibodies, all of the residues in the HCDR2 loop contributed to binding interactions in the single-state but not the multi-state design. In contrast only G63 and T64 residues contributed in the multi-state case but not in single-state designs. Residue L50 was recovered in all single-state complexes but was not critical for multi-state design. For the V_H_3-23 gene, residues A55 and Y66 were not recovered in multi-state design but were found to be important for high affinity in single-state design. For the V_H_5-51 complexes, non-interface residues P15, M53 and A80 were not recovered in multi-state design but were found to be critical in single-state design. HCDR2 was found to be critical in single-state design for all V_H_5-51 complexes.

### Mature Sequence Bias Introduced by Finite Ensemble Size

**Figure 5:**
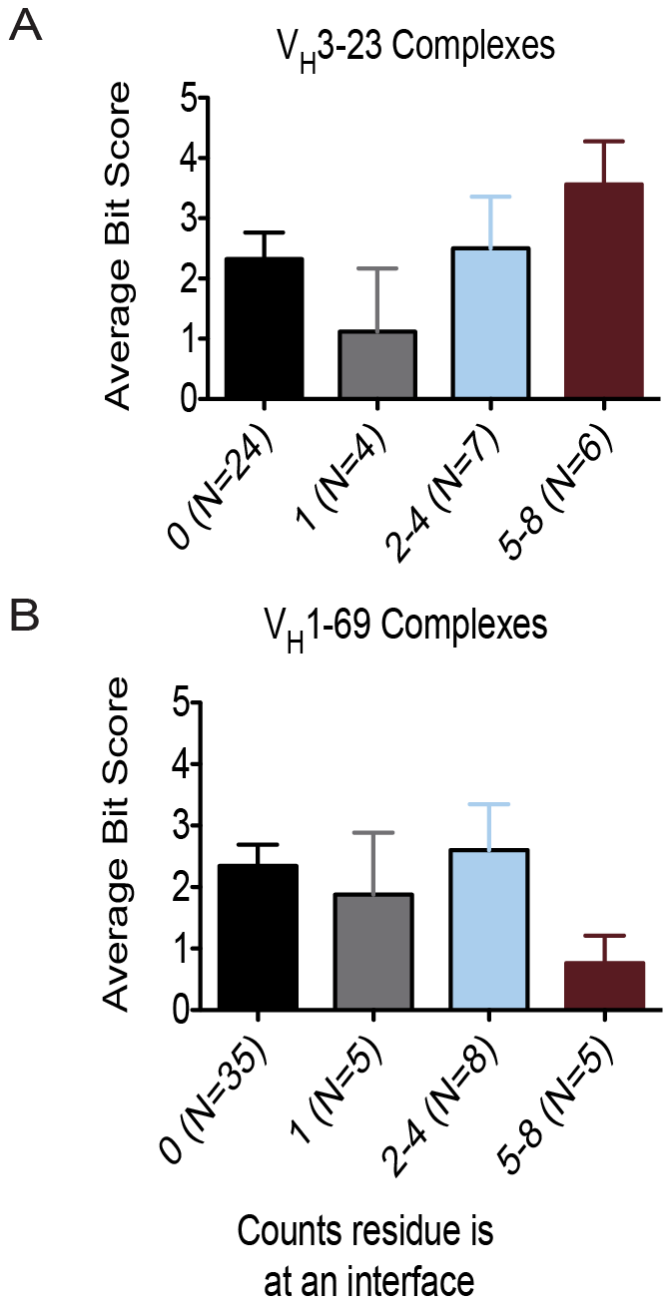
Interface occurrences affect germline sequence recovery. For V_H_3-23 (A) and V_H_1-69 complexes (B), we binned each residue position into how many times it occurred in an interface (interface ensembles). Most designed positions never occurred in an interface. As their occurrences became more frequent, we observed a trend for increasing the recovered germline residue. This trend fell off for V_H_1-69 complexes (B) for positional occurrences between 5–8 interfaces.

To understand some of the trends described above more quantitatively, we determined for each residue in each antibody/antigen complex if it was part of the interface, *i.e.*, directly engaging the antigen. For this purpose the change in neighbor count between unbound antibody and bound antibody/antigen complex score was measured, and positions with a change larger than 1.0 were classified as “interacting residues.” Next, we counted how often a residue position appeared in the interface within each set of antibody/antigen complexes. Positions were binned as occurring in the ensemble interface never, once, two-four times, or more than four times and average bit-scores were compared (Figure 5). We found a general trend for interface ensemble size correlating with interface ensembles sampled. For the set of structures derived from V_H_3-23, which contained a total of 8 complexes, we found that residue positions that are never found in the interface gave an average bit-score of 2.3±0.4. If a residue position was found only in one interface, the average bit-score dropped to 1.2±1.1. As residues were found more frequently at the interface between 2–4 complexes, and 5–8 complexes, the average bit-score increased to 2.5±0.8 and 3.6±0.7 respectively. For the 10 V_H_1-69 complexes, an average bit-score of 2.3±0.3 was observed for residues that were never found in the interface. If a residue was only found in the interface once, the average bit-score dropped to 1.9±1.0. For interface occurrences between 2–4 and 5–8, we found the average bit-score to increase to 2.6±0.7 and drop to 0.8±0.4 respectively. Due to the limited number of residues occurring in multiple interfaces, a significant change in bit-score between each grouping was not observed for V_H_1-69 (p =  0.1844) and V_H_3-23 residue positions (p = 0.2007). We report these findings as an observable trend in ensemble bias as no claims of significance can be made due to the small sample size.

Only the sets of V_H_3-23 and V_H_1-69 gene-encoded antibody/antigen complexes were evaluated, as the set derived from V_H_5-51 had only three structures.

### Evolutionary Sequence Bias Explains in Part Deviations from Germline Sequence for Antibodies Optimal for Polyspecificity

**Figure 6:**
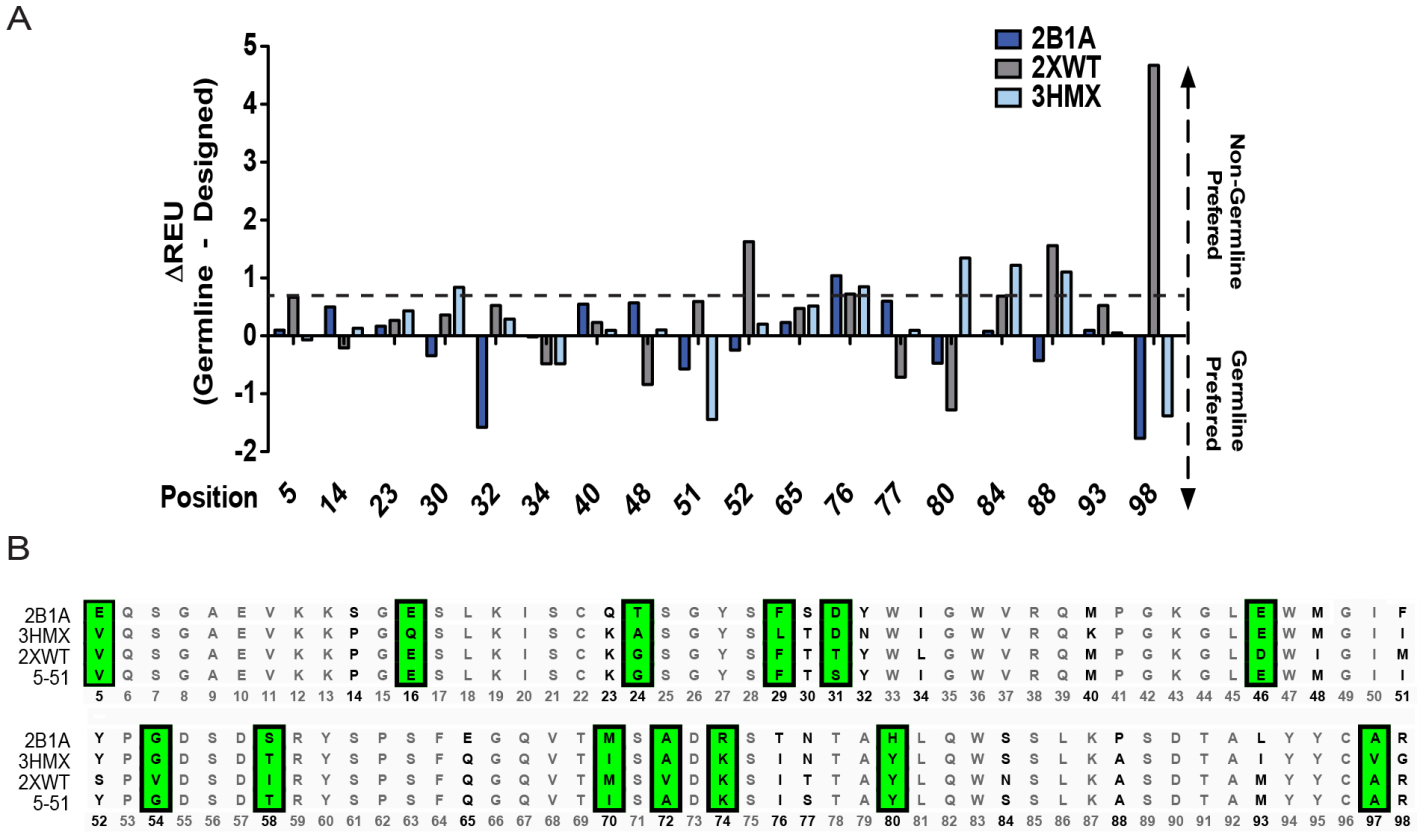
Rosetta multi-state design solutions for non-germline amino acids represent incomplete sampling for V_H_5-51 complexes. We evaluated a complete germline reversion of V_H_5-51 sequence versus the sequences output by multi-state design. (A) Consideration of positions in which the multi-state design algorithm chose a non-germline amino acid for at least 10% of the models where evaluated. The difference in energy of the germline sequence and the multi-state design solution sequence is shown for each position. Bars above 0 represent the multi-state design sequence preferred while bars below the line represent the germline amino acid preference. The horizontal dashed line at 0.7 Rosetta energy units (REU) shows the average energy difference between the germline and mature sequence and is represented as a marker for sequence tolerance. (B) The multiple sequence alignment for each V_H_5-51 complex is shown and compared with the germline sequence. Sequences highlighted in bold were considered for design. Sequences highlighted in green are positions in which the multi-state design algorithm chose the germline amino acid as the design solution. The numbers in the bottom row are the alignment-numbering scheme of each position and correspond to the position numbers in (A).

We expected the result of multi-state design to deviate from germline in cases where alternate amino acids are compatible with the conformational space and binding modes observed in the ensemble of structures. Alternative amino acids might be tolerated but are not observed in evolution – ‘evolutionary sequence bias’. To test this hypothesis, we reverted each position back to germline and compared the energetic change with the favored residue returned by multi-state design. Using reference energies, Rosetta facilitates the direct comparison for energies between different residue types [41]. For complexes derived from V_H_5-51, all positions in which the germline residue was not chosen in at least 10% of the 100 simulated models were forced into the germline identity (Figure 6A, x-axis). The difference in average energy of the germline sequence at that position from the average energy of the residue returned by multi-state design was calculated (y-axis). For each position, if positive values were returned for all three complexes, Rosetta design would most likely place a non-germline amino acid at that position. If negative values were returned for all three complexes, Rosetta would most likely place a germline amino acid at that position. We found that, in most cases, the energetic contribution of the designed amino acid is not significantly more stabilizing than the germline amino acid, i.e. the germline sequence is tolerated as well. Only positions 52, 76, 88, and 98 gave a significant energy increase for the germline sequence in at least one complex. Changes in energy were classified as significant if larger than 0.7 Rosetta energy units (REU, horizontal dashed line). This threshold was derived from the average difference in energy between the germline and mature residue (0.7±0.2 REU, Figure S5). For Figure 6B, a multiple sequence alignment is given as a reference, where each position that was considered in multi-state design is highlighted in bold while each position that recovered well to the germline sequence is highlighted in green.

## Discussion

Germline gene-encoded sequences for commonly used V_H_ segments are hypothesized to possess high conformational flexibility making them ideal for binding diverse antigens, *i.e.*, being polyspecific. During antibody maturation, somatic mutations are introduced that increase affinity for a specific target in part by adding attractive interaction to the antigen (increasing enthalpic gain) and in part by locking the conformation critical for interaction with the specific antigen (reducing entropic cost). Here we tested this hypothesis by analyzing three sets of antibodies, each derived from a commonly used V_H_ gene and each co-crystallized with a protein target in its antigen-specific binding conformation.

We chose to not directly compare conformational flexibility for germline and mature antibodies. While this approach may be feasible in general through predicting the accessible conformational space using molecular dynamics [24], it is challenging to achieve complete sampling of large conformational spaces that include the entire immunoglobulin framework. To circumvent this problem, we chose to solve the inversely related protein design problem, which was to study amino acid sequences that are consistent with the conformational space seen in antibody/antigen co-crystal structures. This approach is complementary and potentially superior as it replaces sampling of the large conformational space in antibody backbone regions with solving the better understood ranking of amino acid sequences, given a certain antibody/antigen complex conformation.

Specifically, we employed multi-state design to find single amino acid sequences that were compatible with the multiple conformations of antigen combining sites. Computational tools to design multi-specific proteins were first described by pioneering work in the Kortemme laboratory [29], [31]. In parallel, Leaver-Fay and colleagues developed a general algorithm for multi-state design in the Rosetta framework, in which they designed one protein to interact with non-native targets [32]. We used the latter tool to design antibody sequences that are optimal for facilitating interactions to 1) multiple and diverse antigens, or 2) a single specific antigen.

In the absence of *a priori* knowledge of the germline or mature sequences or the mechanism of antibody maturation through somatic mutations, multi-state design of one antibody to recognize several target proteins recovered sequences similar to those encoded by the inferred germline gene segment. When designing the same antibody to recognize one specific target, the sequence recapitulated the mature antibody sequence. This trend correlated tightly with the divergence of the mature sequence from the inferred germline sequence, *i.e.*, the more somatic mutations an antibody contained, the more reversions to germline needed in order to facilitate interactions to multiple antigens.

Use of a computational tool to approach questions regarding polyspecificity as a function of protein sequence is advantageous, as the Rosetta design algorithm is able to rapidly enumerate the effect of multiple simultaneous mutations in sequence space for the entire heavy chain variable region. This task is quite difficult if not impossible to complete experimentally at this scale. In this manner, conformational flexibility in the framework regions, CDR1, and CDR2 can be tested in a holistic model. All mutated positions in the V_H_ gene segment were considered simultaneously, including the effect of interactions between different domains in the antibody, thus revealing the role of interface and non-interface residues in both poly- and monospecificity. Because this approach considers multiple antibodies of variable conformation at once, each with a distinct binding mode, the multi-state design algorithm predicts sequences that are inherently flexible and capable of adopting the diverse set of conformations needed to bind to multiple antigens.

Harindranath and colleagues demonstrated that polyspecific antibodies were encoded largely by germline gene sequences [13]. Romesberg and Spiller presented structural evidence for flexibility in germline gene-encoded sequences [16]. In addition, Schmidt *et al.* correlated mature sequence to rigidity of the paratope [30]. Taken together, these data suggest conformational flexibility coupled with pre-sampled conformations of the target binding site as the underlying mechanism for polyspecificity [27]. Here, we used a multi-state design algorithm to assess the contribution of the V_H_ gene segment to specifying an antibody with conformational flexibility, preorganization, and polyspecificity. We found that this property is largely attributed to antibody sequences in the germline gene repertoire, since designing antibodies for polyspecificity, sequences recovered germline gene-encoded sequences, while designing antibodies for monospecificity to a single target, returned sequences similar to the mature antibody. This trend increased in strength the higher the number of somatic mutations that had accumulated, *i.e.*, the further optimized the antibody had become.

Importantly, this effect is not limited to the HCDR3, which often contributes much to antibody specificity. We obtained the same finding to be clearly measurable throughout HCDRs 1 and 2 as well as the immunoglobulin frameworks. We found each germline V_H_ gene to encode a set of amino acids that enabled polyspecificity in a distinct manner. These positions were present not only in the paratope, but also in the buried or semi-buried positions of the immunoglobulin frameworks (Figure 4). Antibody maturation is mediated by a collection of mutations in these positions that refine specificity to the target antigen. The size of the current dataset is limited by the number of antibody-antigen complexes available in the PDB as discussed above (Table 1). We expect, that with an increasing number of antibody-antigen complexes in the PDB it will become easier to discern general trends. However, selected germline sequence positions important for poly-specificity in at least 2 out of the 3 germline test sets were identified: For instance, framework 1 residues 17 and 25, framework 2 residues 40 and 51, and framework 3 residues 70, 71 and 82, all numbered according to IMGT, where found to contribute to polyspecificity. In addition CDR1 residue 35 and CDR2 residues 63 and 64, and CDR3 base residue 105, where also found to be critical for polyspecificity. For all three datasets we found a conserved serine 36 in CDR1 important. These generalities present a possible common molecular mechanism for polyspecificty across germline gene sets. Considering that different framework/interface residues were recovered to their mature sequence for monospecificity, *i.e.* single state design, we can hypothesize from these observations that each antibody develops its own mechanism of monospecificity as a function of the antigen.

We conclude that conformational flexibility in the beta-sheet framework is critical for changing critical regulators of the conformation of the paratope – *i.e.*, the takeoff and landing angles of CDR loops, thereby enabling the paratope of germline antibodies to assume multiple conformations. Accordingly, we find that residues that contribute the most to polyspecificity contain larger deviations of their phi-psi torsion angles (Figure 3). During antibody maturation, mutations in these positions likely lock in the target-specific framework conformation, reducing the entropic cost of target binding. Somatic mutations in the paratope, for example within HCDR1 and HCDR2, can directly increase affinity to a target (enthalpic contribution to free energy), or lock in a conformation that recognizes the target (entropic contribution to free energy). We found that on average 62% of residues in the paratope and 42% of residues in the framework were important for changing the binding pattern of the antibody from polyspecificity to recognition of one specific target (Figure 4).

We recognize several important limitations of our study:

1. We assumed that the Rosetta design protocol determined the optimal sequence for any given design challenge. While it has been demonstrated that Rosetta design typically recovers close-to-optimal sequences [35], inaccuracies in the scoring function and limitations in the sampling algorithm will introduce errors. In the future, this limitation could be reduced by improvements applied to the energy function and comparing the results obtained with complementary energy functions.
2. We assumed that the finite and small set of antibody conformations observed in the set of co-crystallized mature antibodies completely describes the conformational flexibility of the germline gene-encoded antibody (finite ensemble bias). While we used the largest ensembles available (10, 8, and 3 antigen-antibody complexes), this assumption must be wrong, introducing a bias. For example, assume there is a sequence position that is part of the paratope in only one of the *n* complexes. In this antibody, a somatic mutation occurred at this position greatly increasing affinity to the antigen. The somatically mutated amino acid is however compatible with all other *n*-1 complex structures. In such a scenario the multi-state design algorithm will recover the somatically mutated instead of the germline amino acid. Here, we found that as a residue was more often part of the paratope, it became more likely to be recovered to the germline sequence. This finding might be due to the fact that a critical conformation that the germline antibody needs to adopt was not represented in the ensemble (for framework residues) or the epitope needed for recognition by a critical germline amino acid represented in the ensemble.
3. We assumed that the germline gene-encoded antibodies were able to adopt the conformations of each of the mature antibodies derived from it. This assumption is important, as crystal structures of the “true” germline antibody in complex with the antigen are generally not available. While this assumption is expected to be correct for the majority of cases, notable exceptions are discussed in the literature [8], [21], [27], [42].
4. It is not guaranteed that only the germline amino acid is compatible with all conformations adopted by mature antibodies. Rather, it is likely that for some positions alternative amino acids are plausible or even better in realizing the conformational flexibility needed. The germline sequence observed in nature is optimized in evolution and clearly works, but does not need to be perfect in all positions. In such a scenario, multi-state design could return amino acids that deviate from germline (evolutionary sequence bias). Conversely, the mature sequences observed in the co-crystal structures are not guaranteed to be the perfect sequence for high affinity. In some positions a somatic mutation might have introduced a better amino acid but is not the “true” best option. Some somatic mutations might have occurred by chance and do not contribute to affinity maturation. Some positions might not have experienced somatic mutations but still favorable mutations exist. In all these cases we expect the single-state design to deviate from the mature sequence observed in the co-crystal structure (evolutionary sequence bias).
5. The imperfect nature of the Rosetta scoring function will not yield 100% agreement with natural phenomenon [35]. Importantly, water coordination can often be important in antibody-antigen binding sites [43]. However, Rosetta is currently being developed to include tools with explicit solvent models [44].

It is important to understand these biases and limitations to arrive at an accurate interpretation of the results. Given these known limitations, we expected imperfect agreement of *in silico* predicted and natively observed mature and germline gene-encoded antibody sequences. Nevertheless, we found a remarkably high correspondence of residues designed for polyspecificity in a blinded fashion and the amino acids encoded by germline genes.

We identified at least four specific scenarios in which current datasets are limiting for informing design efforts. The first scenario involves a framework position that does not interact with the epitope in any of our tested complexes. For this position, the germline residue, and only the germline residue, is capable of adopting the phi-psi angles in order to accommodate the flexibility needed for the binding site. Multi-state design likely design in the germline residue for each simulation. We then observe agreement between *in silico* design and natively observed sequence for a majority of the designed positions (Figure 2).

The next scenario involves a framework position that also lies distal from the epitope. In this scenario, the germline residue but also other amino acids are compatible with the observed conformations since they both contain properties to adopt the phi-psi angles necessary to accommodate the flexible binding site. For this scenario, we expect Rosetta's multi-state design algorithm to pick one of the compatible amino acids, not necessarily the germline gene-encoded one. This outcome can occur either because the conformational ensemble is incomplete or because of the evolutionary sequence bias. We find that both biases contribute to ambiguity. Residues that are never found in the interface give modest recovery to germline sequences being either “hit-or-miss” (finite ensemble bias, Figure 5), and residues that are reverted to an amino acid different from that encoded in the germline are not significantly better in energy score than the germline encoded amino acid (evolutionary sequence bias, Figure 6).

The third scenario concerns residues that are at part of the paratope in only one instance. If the mature residue forms critical interactions that minimize the free energy of binding in this one complex, while in all other complexes the residue is not part of the paratope and the mature amino acid seen for the one complex is compatible with the backbone confirmation, Rosetta will choose the mature residues from the first complex also in multi-state design mode. We observed this trend, especially for V_H_3-23 complexes. If a residue was found in only one interface (Figure 5), that position tended to have a low recovery to the germline sequence.

The fourth scenario deals with positions that are part of the paratope multiple times and that experience frequent somatic mutations. As positions are found to be more frequently in interface ensembles, the germline recovery increases as these positions become more important to facilitating direct interactions with their antigen (Figure 5). These residues contribute to polyspecificity by being the preferred residue in interaction with multiple antigens, rather than facilitating binding by altering beta-sheet packing.

These results suggest that the naturally occurring antibody maturation process can be recapitulated or reversed at least partially *in silico*, opening exciting new avenues for antibody engineering work. Further, our results suggest the applicability of multi-state design to engineer polyspecific antibodies, exploring another important strategy for designing broadly neutralizing antibody therapeutics. Traditional antibody engineering approaches emphasize isolating monoclonal antibodies that are highly specific for a given antigen, relying on display techniques in which emphasis typically is placed only on CDR loop design. The method described here considers the entire antibody variable region during design, including critical framework residues that allow for conformational flexibility and contribute to polyspecificity. Considering that we found that up to 64% of framework and CDR residues may contribute to binding and specificity, computational design will be invaluable to rapidly enumerate the large sequence and structural space of residues that can contribute to breadth of binding diverse targets.

## Materials and Methods

### Selection of Antigen-Antibody Complexes

Diverse antigen-antibody complexes were collected from the Protein Data Bank (PDB; www.pdb.org) in which antibodies in different complexes were derived from the same predicted heavy chain variable gene segment. Candidate complexes were queried from the protein databank using the IMGT-3D structural query [45] editor for immune system receptors. PDB structures were used as design candidates if they met the following criteria: 1) the antibody was encoded by a V_H_1-69, V_H_3-23, or V_H_5-51 gene segment, 2) the structure contained a human immunoglobulin, and 3) the ligand type was a protein complex. The search yielded 10, 8, or 3 antibody-antigen complexes encoded by the heavy chain variable gene segments V_H_1-69, V_H_3-23, or V_H_5-51, respectively. Nature of the antigen and antibody isotype where not considered in the selection as the 21 complexes represent an exhaustive search of the PDB for these gene-segments. The gene segments were aligned using the ClustalW2 multiple sequence alignment algorithm [46]. Each input structure was energetically minimized using the Rosetta scoring function but constrained to PDB input backbone coordinates [47].

### Multi-state Design of Antigen-Antibody Complexes

Three design experiments were performed, one for each of the three germline segments (V_H_1-69, V_H_3-23, or V_H_5-51) using the multi-state design mode of the Rosetta algorithm and scoring functions. We adapted a generalized multi-state design protocol that was described in detail previously to perform design on multiple antibody-antigen complexes at once (Figure 1) [32]. Briefly, each computational design experiment computed an optimal sequence predicted to define a low-energy structure. In the multi-state design experiments, an energetic consensus sequence for all of the states was predicted, rather than treating each state as a separate entity. The energy for a given sequence was computed and designated the ‘*design fitness’* for all states. The corresponding amino acids were derived from the alignment (*e.g*., heavy chain amino acid 5 on complex A corresponded to heavy chain amino acid 5 on complex B). The details of the multi-state algorithm is described elsewhere [32].

### Single-State Design of Antigen-Antibody Complexes

Single-state design was performed using the Rosetta multi-state application where the algorithm was altered so that only one complex was considered for each of the 10, 8, or 3 design experiments with V_H_1-69, V_H_3-23, or V_H_-69 complexes, respectively.

### Design Analysis of Multiple- or Single-State Design

For each design experiment, 100 independent design trajectories were calculated. Sequence logos then were generated using the Berkley web-logo server (http://weblogo.berkeley.edu/) [36]. Information for each sequence logo can be extrapolated as follows extending the work of Schneider *et al.* [38]. For each variable position, the probability of seeing each of the 20 naturally encoded amino acids p_i_ was computed and compared with the background probability p_b_ = 1/20 = 5%. To quantify the deviation of the observed probability from the background probability we compute the self-information for each of the 20 amino acids as I_i_ = p_i_ x log_2_(20 x p_i_) in ‘bit’. If the amino acid occurs as often as expected from the background probability, I_i_ is zero. I_i_ becomes larger if the amino acid is over-represented and approaches 4.32 if p_i_ = 100%. A total bit-score for the sequence design was obtained by summing all individual bit-scores for each amino acid (Figures 1D,S3). The bit-scores for the target sequence then were analyzed, and statistics were computed using Prism software version 5.0 (GraphPad Software). For comparisons between germline sequence and mature sequence within the same design experiment, a Wilcoxon matched pairs test (non-normal, paired t-test) was used to compute the p-value at 99% confidence level. For comparison between design experiments, a student's paired t-test was used to compute the p-value at 99% confidence level.

### Amino Acid Environment

The neighbor vector algorithm quantitatively determines the surface-exposure of a given residue and is described by Durham and colleagues elsewhere [48]. Briefly, each C_β_ is computed to a vector and each vector is given a score based on the number and orientation of each C_β_ in the proximity. The weight of each neighbor falls of as a function of distance.

For interface scores, the change in neighbor vector was used, where the neighbor vector score of the amino acids in the unbound antibody is subtracted from the neighbor vector scores of the complex. Interface residues would have a large change in neighbors and proportional to the change in neighbor vector score.

### Phi-psi Angle Calculations

All V_H_ framework residues were grouped by complex. For each residue, phi-psi angles and secondary structure classification were determined using dssp2 [49]. For each residue position across all complexes considered in design, the standard deviation of the phi-psi angles was calculated if they were included in the beta-sheet framework. A student's t-test was performed between the standard deviations between residue positions that recovered to germline (bit-score>1), or did not recover to germline (bit-score<1). For a reference, a deviation for all framework beta-sheet positions was also calculated for all residues even if they were not included in the design protocol.

### Protocol Capture

A workable example for V_H_5-51 complexes using multi-state design including analysis scripts can be found at ww.meilerlab.org/index.php/jobs/resources under the protocol capture tag. The Rosetta Modeling Suite can be obtained at www.rosettacommons.org

## Supporting Information

Figure S1 **Sequence alignments of test set sequences to their inferred germline.** Multiple sequence alignments of each of the antibodies mature V_H_ genes to the germline sequence. Designed positions that differ in at lease one complex from the germline are shown in black, while conserved residues are grayed out. The locations of the frameworks (FR1, 2, or 3) and complementarity determining regions are annotated. (A) IGV_H_1-69 derived complexes with the germline sequences for the allelic variants labeled as 6901, 6902, 6905, and 6910. (B) IGV_H_3-23 derived complexes with allelic variants labeled 2301 and 2304. (C) IGV_H_5-51 complexes with only one variant labeled 5–51.
(PDF)

Figure S2 **Comparison of designed residues with sequence logos for multi-state design.** Each of the designed residues propensities is shown as a sequence logo. The germline sequence and the mature sequence of each of the complexes are shown below each logo. Amino acid sequences that match the logo are shown in black while sequences that did not match the logo are shown in grey. For multi-state design, more residues of the sequence logo match the germline sequence for (A) IGV_H_1-69 complexes, (B) IGV_H_3-23 complexes and (C) IGV_H_5-51 complexes.
(PDF)

Figure S3 **Total and individual bit scores for each multi- and single-state design.** (A) The average bit score for each complex as a function of how well it matched the germline or mature sequence. The change in bit score is the difference for the proclivity for multiple complexes in multi-state design to design for either the mature or germline sequence, positive and negative numbers respectively. (B) Individual complex scores decomposed from the sums of (A).
(PDF)

Figure S4 **Proclivity to mature or germline sequences is a function of design protocol and degree of maturation.** (A) The change in bit score is determined to be the proclivity to either the mature (positive score) or the germline (negative score) sequence. For these calculations only complexes with greater than 12 somatic mutations in the variable gene are considered and assigned a change in bit score. The change in bit score is plotted as a function of mutations either from the germline sequence (single-state) or from the mature sequence (multi-state). This *in silico* maturation (single-state) or reversion (multi-state) correlates with the number of mutations with r^2^ = 0.8964. (B) Only highly mutated antibody-antigen complexes (>12 mutations away from germline) are summed for single- and multi-state design with a more significant change in bit score.
(PDF)

Figure S5 **Recovered germline sequences are not an energetic consensus.** We reverted somatically mutated amino acids back to their inferred germline gene-encoded sequence and compared each individual per-amino-acid energy to that of the mature sequence per-amino acid energy. (A) Mutations are binned into three categories: (1) amino acids that had somatically mutated away from germline in which the multi-state design algorithm recovered the germline encoded residue as the preferred residue to enable polyspecificity, (2) amino acids somatically mutated away from germline in which multi-state design chose an amino acid other than the germline or mature sequence as the preferred residue to enable polyspecifity, and (3) as a control group, amino acids that remained germline in the mature complex. The energies for each germline encoded amino acid were subtracted from the energy of the somatically mutated amino acid. We found that the mutations reverted back to germline by multi-state design lost an average energy of 0.9±1.2 Rosetta energy units (REU). We also examined mutations where the germline amino acid was not chosen in multi-state design and looked at the change in energy if a germline amino acid sequence was forced at that position. As expected, if the multi-state design protocol would have chosen a germline amino acid at that position, the average energy each mutation would lose is 0.7±1.4 REU, which was not a statistically significant change from recovered germline mutations (p = 0.5). This observation suggests that a mutation from the germline gene-encoded amino acid was judged equally beneficial in these cases by the computational protocol but another amino acid was identified that was equally compatible with the conformations observed in the ensemble. For a control experiment, we examined the energies of sequences that did not mutate. We found that the energy difference for non-mutated amino acids between the germline structure and the mature structure was close to zero (0.0±0.1). The energies examined for changes from germline to mature differed significantly from non-mutations (p = 0.0041). We also noted that the proclivities for germline or mature sequences were spread across the entire immunoglobulin fold. (B) Surface display maps each change in energy between the germline and mature sequence as a heat map onto the antibody complex.
(PDF)
