# Supplementary material for: Human Germline Antibody Gene Segments Encode Polyspecific Antibodies
Source: PLoS Comput Biol. 2013 Apr 25;9(4):e1003045. doi: 10.1371/journal.pcbi.1003045 (PMC3636087; doi:10.1371/journal.pcbi.1003045)
Supplement: Figure S1 — Sequence alignments of test set sequences to their inferred germline. Multiple sequence alignments of each of the antibodies mature VH genes to the germline sequence. Designed positions that differ in at lease one complex from the germline are shown in black, while conserved residues are grayed out. The locations of the frameworks (FR1, 2, or 3) and complementarity determining regions are annotated. (A) IGVH1-69 derived complexes with the germline sequences for the allelic variants labeled as 6901, 6902, 6905, and 6910. (B) IGVH3-23 derived complexes with allelic variants labeled 2301 and 2304. (C) IGVH5-51 complexes with only one variant labeled 5–51. (PDF) [file pcbi.1003045.s001.pdf]

S1-A

```
PDB ID                FR 1                      CDR 1          FR 2             CDR 2
1G9M   QVQLLESGAEVKKPGSSVKVSCKASGDTFIRYSFTWVRQAPGQGLEWMGRIITILDVAHY
2CMR   --QLVQSGAEVRKPGASVKVSCKASGDTFSSYAISWVRQAPGQGLEWMGGIIPIFGTANY
2DD8   -VQLQQSGAEVKKPGSSVKVSCKASGGTFSSYTISWVRQAPGQGLEWMGGITPILGIANY
2XRA   --QLVESGAEVKKPGSSVKVSCKTSGGTFNRLAMSWVRQAPGQGLEWMGGIMPIFDITNY
2XTJ   QVQLVQSGAEVKKPGSSVKVSCKASGGTFNSHAISWVRQAPGQGLEWMGGINPILGIANY
3FKU   QVQLVQSGAEVKKPGSSVKVSCTSSEVTFSSFAISWVRQAPGQGLEWLGGISPMFGTPNY
3GBM   EVQLVESGAEVKKPGSSVKVSCKASGGPFRSYAISWVRQAPGQGPEWMGGIIPIFGTTKY
3MA9   --QLVQSGAEVKKPGSSVKVSCKASGGTFNSYAFSWVRQAPGQGLEWMGSIIPIFGTTNY
3MAC   --QLVQSGAEVKKPGSSVKVSCKASGGTFNSYAFSWVRQAPGQGLEWMGSIIPLFGFVVY
3P30   ALQLVQSGAEVKKAGSSVRVSCKASGATFSSYSISWVRQAPGQGPQWMGGIVPSSGAAKY
6901   QVQLVQSGAEVKKPGSSVKVSCKASGGTFSSYAISWVRQAPGQGLEWMGGIIPIFGTANY
6902   QVQLVQSGAEVKKPGSSVKVSCKASGGTFSSYTISWVRQAPGQGLEWMGRIIPILGIANY
6905   QVQLVQSGAEVKKPGSSVKVSCKASGGTFSSYAISWVRQAPGQGLEWMGGIIPIFGTANY
6910   QVQLVQSGAEVKKPGSSVKVSCKASGGTFSSYAISWVRQAPGQGLEWMGGIIPILGIANY
         ** :*****:*.*:**:***.:*  .*   :::***** *** :*:* * .  .   *

1G9M   APHLQGRVTITADKSTSTVYLELRNLRSDDTAVYFCAG
2CMR   AQAFQGRVTITANESTSTAYMELSSLRSEDTAIYYCAR
2DD8   AQKFQGRVTITTDESTSTAYMELSSLRSEDTAVYYCAR
2XRA   AQKFQGRVTIITDESTSTAYMELRSLTSEDSAVYYCAR
2XTJ   AQKFQGRVTITADESTSTAYMELSSLRSEDTAVYYCAR
3FKU   AQKFQGRVTITADQSTRTAYMDLRSLRSEDTAVYYCAR
3GBM   APKFQGRVTITADDFAGTVYMELSSLRSEDTAMYYCAK
3MA9   AQKFQGRVTITADESTSTAYMELSSLRSEDTAVYYCAR
3MAC   AQKFQGRVTITADESTSTAYMELSSLRSEDTAVYYCAR
3P30   AQQFQGRLTITADTSTNTAYLELSSLRYDDTAVYYCTR
6901   AQKFQGRVTITADESTSTAYMELSSLRSEDTAVYYCAR
6902   AQKFQGRVTITADKSTSTAYMELSSLRSEDTAVYYCAR
6905   AQKFQGRVTITTDESTSTAYMELSSLRSEDTAVYYCAR
6910   AQKFQGRVTITADKSTSTAYMELSSLRSEDTAVYYCAR
       *  :***:** ::  : *.*::* .*  :*:*:*:*:
                         FR 3
```

S1-B

```
PDB ID                FR 1                      CDR 1          FR 2             CDR 2
1S78   EVQLVESGGGLVQPGGSLRLSCAASGFTFTDYTMDWVRQAPGKGLEWVADVNPNSGGSIY
2FJG   EVQLVESGGGLVQPGGSLRLSCAASGFTISDYWIHWVRQAPGKGLEWVAGITPAGGYTYY
2QQN   EVQLVESGGGLVQPGGSLRLSCAASGFTFSSYAMSWVRQAPGKGLEWVSQISPAGGYTNY
2VYR   EVQLLESGGGLVQPGGSLRLSCAASGFTFEEYAMLWVRQAPGKGLEWVSGINARGYTTYY
2R56   QVSLRESGGGLVQPGRSLRLSCTASGFTFRHHGMTWVRQAPGKGLEWVASLSGSGTKTHF
3KR3   EVQLLESGGGLVQPGGSLRLSCAASGFTFSNYIMWWVRQAPGKGLEWVSVISSSGGMTRY
3BN9   QVQLVQSGGGLVKPGGSLRLSCAASGFTFSSYAMSWVRQAPGKGLEWVSAISGSGGSTYY
3DVN   EVQLVESGGGLVQPGGSLRLSCAASGFNVKTGLIHWVRQAPGKGLEWVAYISPYYGSTSY
2301   EVQLLESGGGLVQPGGSLRLSCAASGFTFSSYAMSWVRQAPGKGLEWVSAISGSGGSTYY
2304   EVQLVESGGGLVQPGGSLRLSCAASGFTFSSYAMSWVRQAPGKGLEWVSAISGSGGSTYY
       :*.* :******:** ******:****..    : *************: :.     : :

1S78   NQRFKGRFTLSVDRSKNTLYLQMNSLRAEDTAVYYCAR
2FJG   ADSVKGRFTISADTSKNTAYLQMNSLRAEDTAVYYCAR
2QQN   ADSVKGRFTISADTSKNTAYLQMNSLRAEDTAVYYCAR
2VYR   ADSVKGRFTISRDNSKNTLYLQMNSLRTEDTAVYYCAK
2R56   ADSVKGRFTISRDNSNNTLYLQMDNVRDEDTAIYYCAK
3KR3   ADSVKGRFTISRDNSKNTLYLQMNSLRAEDTAVYYCAR
3BN9   ADSVKGRFTISRDNSKNTLYLQMSSLRAEDTAVYYCAR
3DVN   ADSVKGRFTISADTSKNTAYLQMNSLRAEDTAVYYCAR
2301   ADSVKGRFTISRDNSKNTLYLQMNSLRAEDTAVYYCAK
2304   ADSVKGRFTISRDNSKNTLYLQMNSLRAEDTAVYYCAK
        : .*****:* * *:** ****..:* ****:****:
                         FR 3
```

S1-C

```
PDB ID                 FR 1                      CDR 1          FR 2            CDR 2

2XWT    EVQLVQSGAEVKKPGQSLKISCKASGYSLTDNWIGWVRQKPGKGLEWMGIIYPGDSDTRY
3HMX    EVQLVQSGAEVKKPGESLKISCKGSGYSFTTYWLGWVRQMPGKGLDWIGIMSPVDSDIRY
2B1A    EIQLEQSGAEVKKSGESLKISCQTSGYSFSDYWIGWVRQMPGKGLEWMGIFYPGDSDSRY
5-51    EVQLVQSGAEVKKPGESLKISCKGSGYSFTSYWIGWVRQMPGKGLEWMGIIYPGDSDTRY
        *:** ********.*:******: ****::  *:***** *****:*:**: * *** **

2XWT    SPSFQGQVTISADKSINTAYLQWSSLKASDTAIYYCVG
3HMX    SPSFQGQVTMSVDKSITTAYLQWNSLKASDTAMYYCAR
2B1A    SPSFEGQVTMSADRSTNTAHLQWSSLKPSDTALYYCAR
5-51    SPSFQGQVTISADKSISTAYLQWSSLKASDTAMYYCAR
        ****:****:*.*:* .**:***.***.****:***.

                         FR 3
```
