# Supplementary figures and images for: Human Germline Antibody Gene Segments Encode Polyspecific Antibodies
Source: PLoS Comput Biol. 2013 Apr 25;9(4):e1003045. doi: 10.1371/journal.pcbi.1003045 (PMC3636087; doi:10.1371/journal.pcbi.1003045)

S2-A

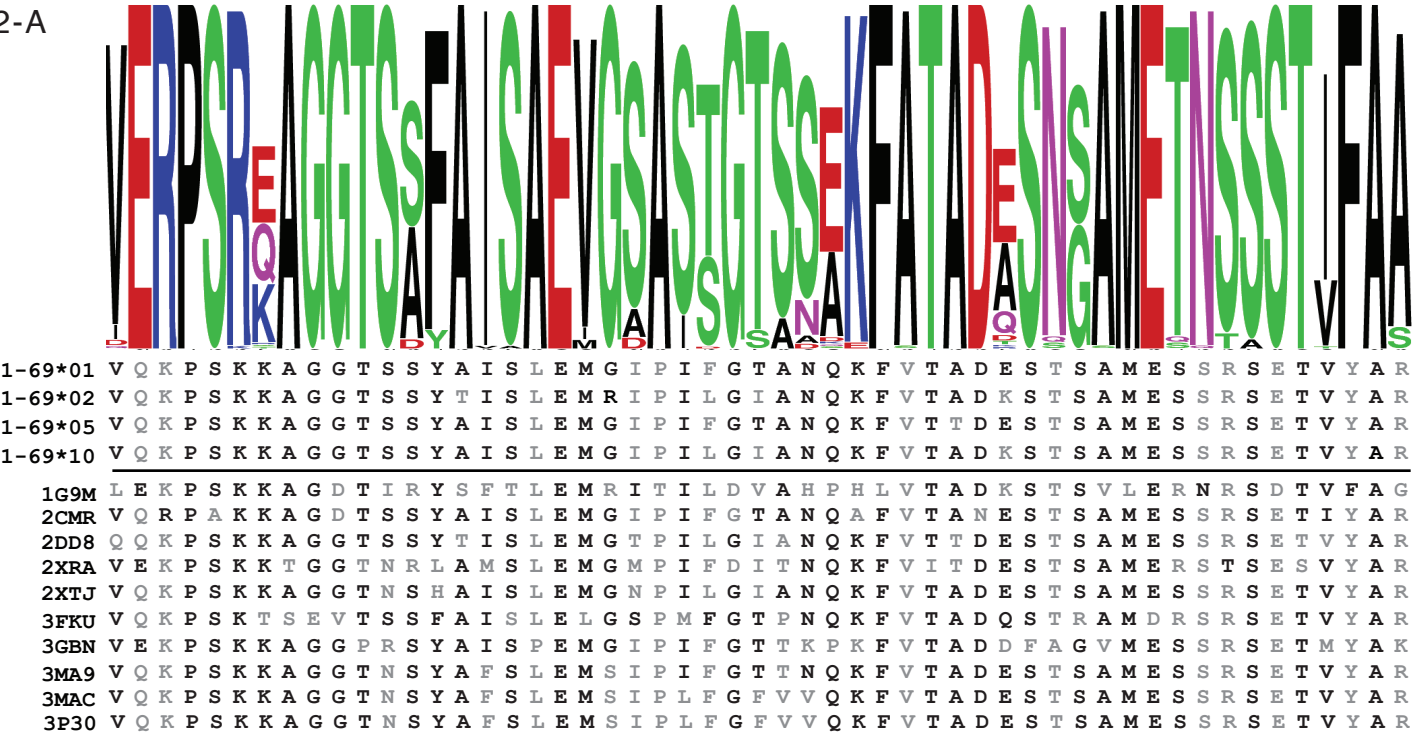

S2-B

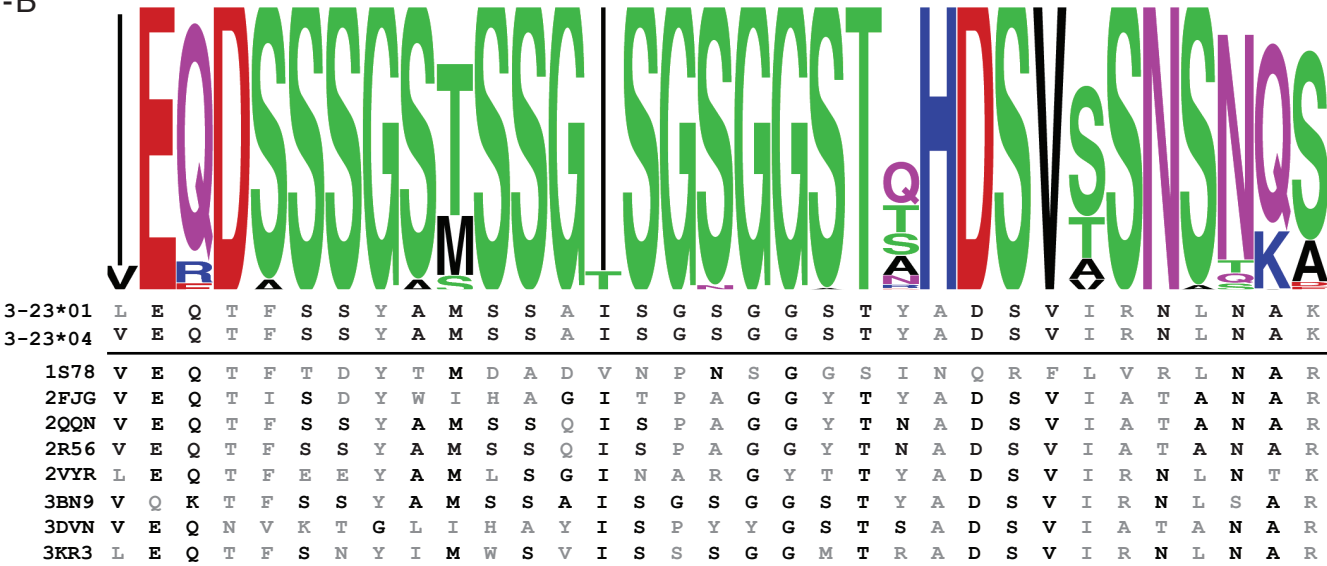

S2-C

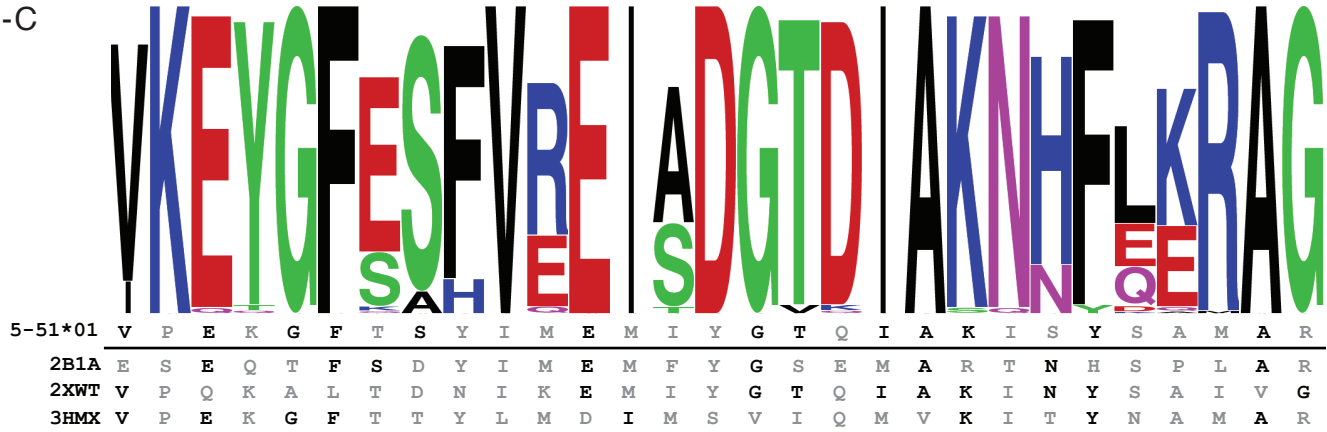

Supplement: Figure S2 — Comparison of designed residues with sequence logos for multi-state design. Each of the designed residues propensities is shown as a sequence logo. The germline sequence and the mature sequence of each of the complexes are shown below each logo. Amino acid sequences that match the logo are shown in black while sequences that did not match the logo are shown in grey. For multi-state design, more residues of the sequence logo match the germline sequence for (A) IGVH1-69 complexes, (B) IGVH3-23 complexes and (C) IGVH5-51 complexes. (PDF) [file pcbi.1003045.s002.pdf]

S4-A

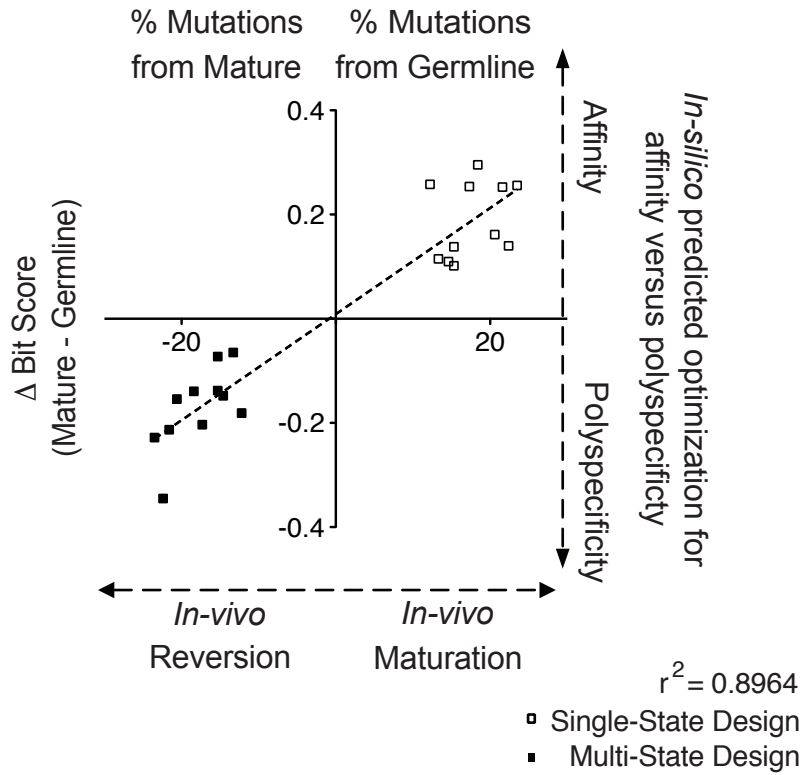

S4-B

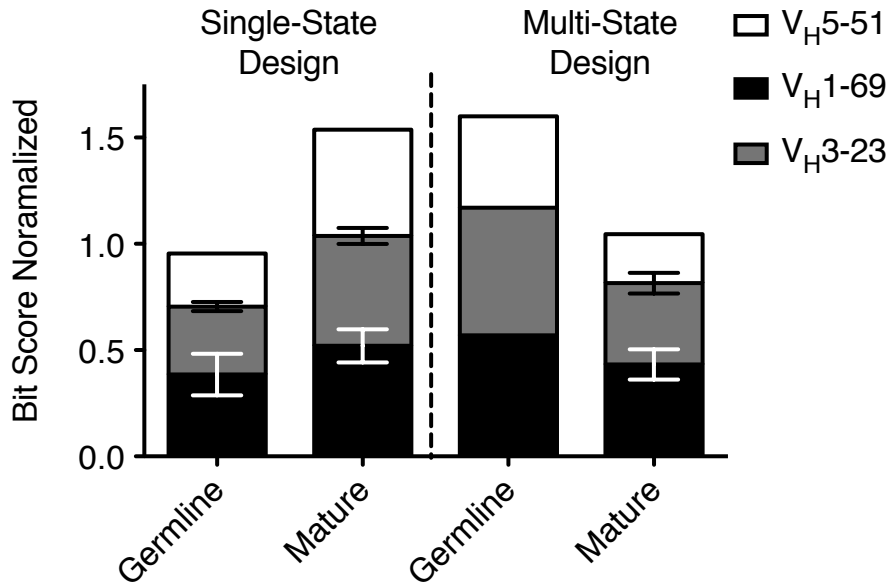

Supplement: Figure S4 — Proclivity to mature or germline sequences is a function of design protocol and degree of maturation. (A) The change in bit score is determined to be the proclivity to either the mature (positive score) or the germline (negative score) sequence. For these calculations only complexes with greater than 12 somatic mutations in the variable gene are considered and assigned a change in bit score. The change in bit score is plotted as a function of mutations either from the germline sequence (single-state) or from the mature sequence (multi-state). This in silico maturation (single-state) or reversion (multi-state) correlates with the number of mutations with r2 = 0.8964. (B) Only highly mutated antibody-antigen complexes (>12 mutations away from germline) are summed for single- and multi-state design with a more significant change in bit score. (PDF) [file pcbi.1003045.s004.pdf]

S5-A

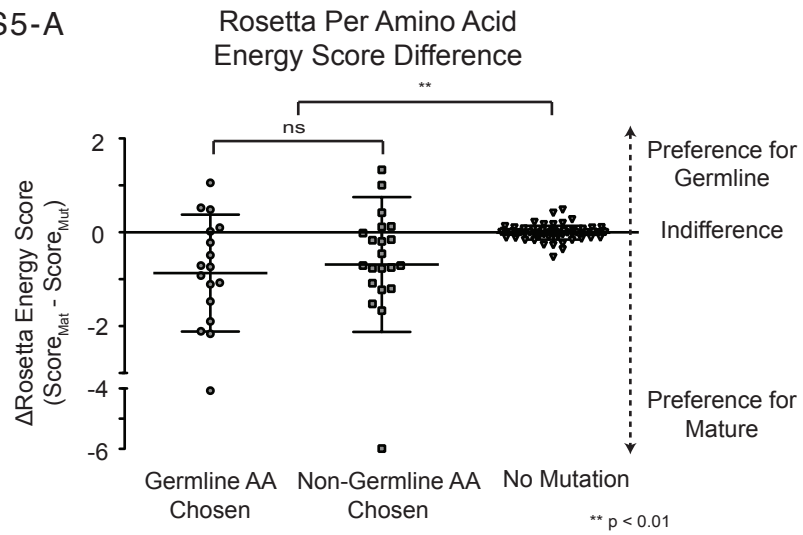

S5-B

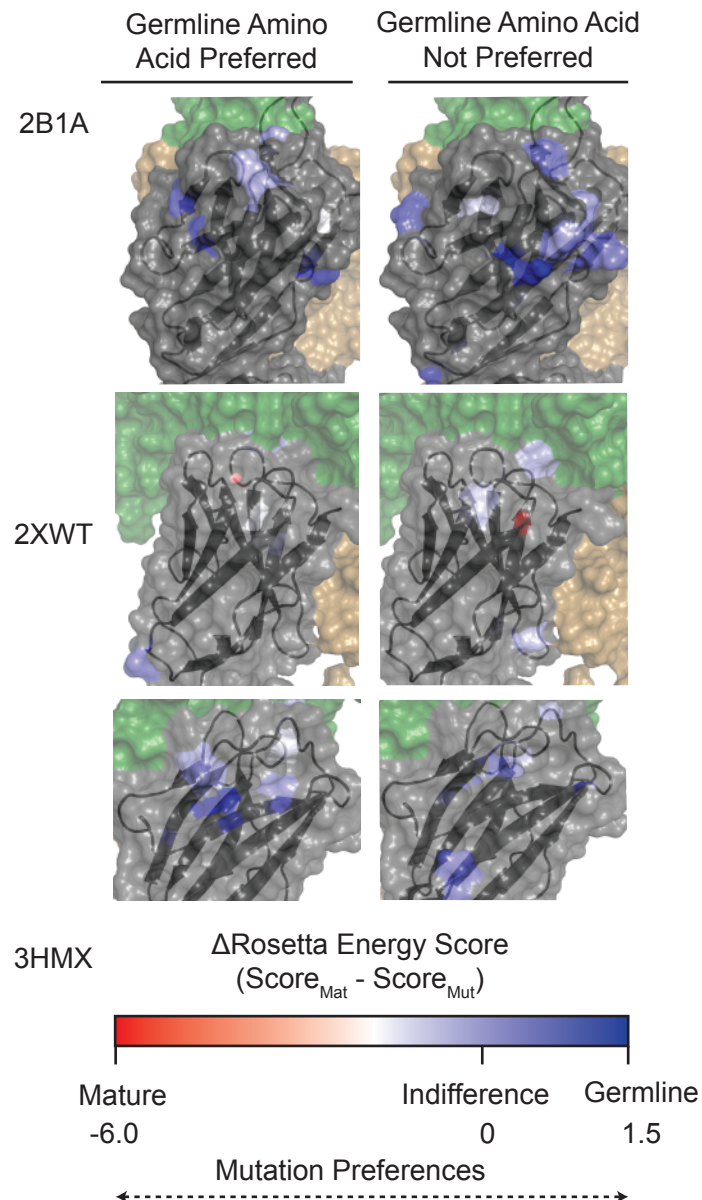

Supplement: Figure S5 — Recovered germline sequences are not an energetic consensus. We reverted somatically mutated amino acids back to their inferred germline gene-encoded sequence and compared each individual per-amino-acid energy to that of the mature sequence per-amino acid energy. (A) Mutations are binned into three categories: (1) amino acids that had somatically mutated away from germline in which the multi-state design algorithm recovered the germline encoded residue as the preferred residue to enable polyspecificity, (2) amino acids somatically mutated away from germline in which multi-state design chose an amino acid other than the germline or mature sequence as the preferred residue to enable polyspecifity, and (3) as a control group, amino acids that remained germline in the mature complex. The energies for each germline encoded amino acid were subtracted from the energy of the somatically mutated amino acid. We found that the mutations reverted back to germline by multi-state design lost an average energy of 0.9±1.2 Rosetta energy units (REU). We also examined mutations where the germline amino acid was not chosen in multi-state design and looked at the change in energy if a germline amino acid sequence was forced at that position. As expected, if the multi-state design protocol would have chosen a germline amino acid at that position, the average energy each mutation would lose is 0.7±1.4 REU, which was not a statistically significant change from recovered germline mutations (p = 0.5). This observation suggests that a mutation from the germline gene-encoded amino acid was judged equally beneficial in these cases by the computational protocol but another amino acid was identified that was equally compatible with the conformations observed in the ensemble. For a control experiment, we examined the energies of sequences that did not mutate. We found that the energy difference for non-mutated amino acids between the germline structure and the mature structure was close to zero ( [file pcbi.1003045.s005.pdf]
