# Supplementary material for: Human Germline Antibody Gene Segments Encode Polyspecific Antibodies
Source: PLoS Comput Biol. 2013 Apr 25;9(4):e1003045. doi: 10.1371/journal.pcbi.1003045 (PMC3636087; doi:10.1371/journal.pcbi.1003045)
Supplement: Figure S3 — Total and individual bit scores for each multi- and single-state design. (A) The average bit score for each complex as a function of how well it matched the germline or mature sequence. The change in bit score is the difference for the proclivity for multiple complexes in multi-state design to design for either the mature or germline sequence, positive and negative numbers respectively. (B) Individual complex scores decomposed from the sums of (A). (PDF) [file pcbi.1003045.s003.pdf]

S3-A

	Multi-State Design			Single-State Design		
	Germline	Mature	ΔBit Score (Mature-Germline)	Germline	Mature	ΔBit Score (Mature-Germline)
V_H1-69	0.54	0.48	-0.06	0.46	0.57	0.11
V_H3-23	0.60	0.45	-0.14	0.43	0.54	0.11
V_H5-51	0.43	0.26	-0.18	0.28	0.53	0.25

S3-B

	Multi-State Design			Single-State Design		
	Germline	Mature	ΔBit Score (Mature-Germline)	Germline	Mature	ΔBit Score (Mature-Germline)
1G9M	0.54	0.36	-0.18	0.37	0.60	0.24
2CMR	0.54	0.52	-0.02	0.43	0.46	0.03
2DD8	0.54	0.49	-0.05	0.54	0.60	0.06
2XRA	0.54	0.42	-0.12	0.38	0.50	0.12
2XTJ	0.54	0.53	-0.01	0.47	0.56	0.09
3FKU	0.54	0.51	-0.04	0.43	0.57	0.14
3GBN	0.54	0.50	-0.04	0.51	0.68	0.17
3MA9	0.54	0.51	-0.03	0.57	0.64	0.07
3MAC	0.54	0.49	-0.05	0.48	0.59	0.11
3NPS	0.54	0.57	0.03	0.61	0.63	0.02
3P30	0.54	0.42	-0.13	0.25	0.41	0.17
1S78	0.60	0.25	-0.35	0.28	0.42	0.14
2FJG	0.60	0.46	-0.14	0.38	0.48	0.10
2QQN	0.60	0.52	-0.08	0.56	0.60	0.05
2R56	0.60	0.37	-0.23	0.32	0.58	0.26
2VXS	0.60	0.60	0.00	0.54	0.54	0.00
2VYR	0.60	0.42	-0.18	0.51	0.62	0.11
3BN9	0.60	0.52	-0.08	0.45	0.42	-0.03
3DVN	0.60	0.46	-0.14	0.29	0.59	0.30
3KR3	0.60	0.49	-0.10	0.52	0.62	0.11
2B1A	0.43	0.23	-0.20	0.25	0.50	0.25
2XWT	0.43	0.29	-0.14	0.35	0.59	0.24
3HMX	0.43	0.25	-0.18	0.25	0.51	0.26
